# Progress in Psychiatry

**Published:** 1904-04-16

**Authors:** 


### PROGRESS IN PSYCHIATRY.

**Acute Alcoholic Psychoses.**—The investigations of cerebral changes in sub-acute and chronic alcoholism by Andriezen in this country (with the use of Golgi's and Nissl's methods),[1] and by Berkley in America (with the same methods),[2] combined with more recent researches by Ewing,[3] have thrown light on the nature of the pathological process occurring in sub-acute and chronic alcoholic insanity which will be described below. Cases of delirium tremens of alcoholic origin seldom end fatally if the subject is free from cardiac, arterial, renal, or pulmonary disease. Two interesting cases are, however, recorded by Ewing.[4] The first case was that of a young man aged 25 years, who drank heavily and continued practically in a state of intoxication for six weeks, when an attack of delirium tremens supervened with pyrexia and profound physical exhaustion. The temperature rose to 104° F. before death. The second case was that of a man aged 29 years, who, as the result of similar alcoholic indulgence over a period of three months (12 weeks), developed delirium tremens and died with a temperature of 105° F. Post-mortem examinations were made six and twelve hours respectively after death in the above two cases, and the following pathological changes were found. There was intense and general capillary congestion of the brain of both subjects. Sections of the brain, medulla, and spinal cord were prepared after hardening in Lang's fluid and staining with Nissl's methylene-blue method. The changes revealed were both striking and instructive. To begin with, the large multipolar "motor" cells of the cord and bulb were extensively affected and were nowhere normal. The chromatin bodies—the normal stored-up pabulum of the nerve-cells which is used up during functional activity—were almost entirely disintegrated and destroyed, or were absent, in these motor cells. In many other cells this "chromatolysis" was even more advanced, and the cell-body showed transparent areas of hyaline degeneration. Further, the outlines of many cells were irregular and ragged (surface erosion), and the nuclei were dislocated from their normal position, sometimes so markedly as to project from the side of the cell-body. Similar chromatolytic changes, but to a less extent, were found in Purkinje's cells of the cerebellum. In the cerebral hemispheres the cortical pyramidal cells failed to exhibit the usual reticulated arrangement of the Nissl's bodies, and those Nissl's bodies which were present were either feebly coloured and indistinct or broken up into coarse granules. In the second of the two patients these changes were more marked than in the first. Dr. Ewing compares these changes in the nerve-cells of the brain, cerebellum, and bulbo-spinal cord in delirium tremens with the changes produced experimentally by acute alcoholic poisoning in animals, and declares that the degenerative and destructive changes in delirium tremens are more marked. The nervous symptoms of delirium tremens—restlessness, tremor, insomnia, and pyrexia before death—are attributed to the profound changes of a destructive character taking place in the central nervous system, as shown in the above cases. Important observations on the clinical and etiological aspects of delirium tremens have been recently published by Jacobsen of Copenhagen (based on a study of 124 cases), Villiers of Brussels (study of 100 cases at St. John's Hospital, Brussels), Hertz, a Danish physician, an abstract of whose work is published in the *Journal of Mental Science* of April 1899), Evensen, and Bonhoffer.[5] The following are the conclusions reached: 1. As regards age there is nothing specific to note; it may vary from 25 to 90 years. 2. All the patients were chronic alcoholics, whose beverage consisted of beer, wine, or spirits. It was generally found that the quantity of alcohol (calculated from its percentage quantity in the liquor consumed) necessary to produce or favour the development of delirium tremens was about half a pint a day. 3. Though fever was a common occurrence, yet it was not an invariable accompaniment of the delirium. 4. The commonest visceral troubles accompanying the attack were catarrhal pneumonia and subacute or acute nephritis. Cardiac disease or phthisis, if present, accelerated the course of the symptoms and made the prognosis grave. 5. Albuminuria occurred in a varying proportion of cases—viz. 16 per cent. (Villiers) to 60 per cent. (Jacobsen). Dr. Hertz's observations, on the other hand, tended to show that "uncomplicated delirium tremens was always accompanied by an acute disturbance of the renal functions (nephritis), with accompanying albuminuria." This acute nephritis was an early disturbance immediately preceding the delirium. The albuminuria of delirium tremens was always temporary, as observed in all of Jacobsen's and Villiers' cases. Its permanence would have indicated organic renal disease, which therefore must be regarded as absent in these cases. 6. Loss of weight (from a few ounces to about a stone) occurs during the illness. 7. Insomnia is a frequent and distressing symptom. Thus, of Dr. Villiers' patients 26 suffered from insomnia for two days, 39 for three days, 22 for four days, and 10 for five days. 8. The severity of the disease and the mortality varied considerably. Dr. Villiers' cases in Brussels showed a mortality of 1·5 per cent., while Jacobsen's cases at Copenhagen had a death-rate of 13 per cent. Delirium tremens is apparently milder and attended with fewer complications in the population of Brussels. The explanation of this lies not so much in the racial factor as in the bodily factor—viz. the constitutional condition of the drinker at the time of onset of the delirium. "The Germans who have drunk beer from their youth suffer more frequently from enlarged stomach, fatty heart, and damaged kidneys, and would suffer more severely therefore from delirium tremens after indulging in a drinking debauch." 9. It is concluded that delirium tremens is due to an acute cerebral toxæmia, the combined result of insufficient renal elimination, auto-intoxication from the disordered digestive tract, and alcoholic poisoning of the nervous tissues. The special form which it takes—viz. hallucinatory and terrifying visions, tremor, wasting, and rapid though temporary physical and mental breakdown—is attributed to its occurring, as it almost invariably does, in subjects debilitated by habits of previous and frequent alcoholic indulgence.

[1] Brain, winter 1894. [2] Brain, spring 1895. [3] Arch. of Neurology and Psycho-pathology, 1899, Vol. I. [4] Ibid. [5] Vide Lancet, July 15, 1899, p. 169, and Monatschrifft für Psychiat. und Neurol., 1900.

(*To be continued.*)

